# Clinical Effectiveness of Vestibular Shields in Orthodontic Treatment: A Scoping Review

**DOI:** 10.3390/children10010016

**Published:** 2022-12-22

**Authors:** Chaypat Simsuchin, Yong Chen, Sreekanth Kumar Mallineni

**Affiliations:** 1 Department of Orthodontic and Orthopedic Craniofacial Growth Modification, Faculty of Dentistry, Nation University, Chiang Mai 50210, Thailand; 2 Department of Stomatology, School of Medicine, Xiamen University, Xiamen 361005, China; 3 Center for Transdisciplinary Research (CFTR), Saveetha Dental College, Saveetha Institute of Medical and Technical Sciences, Saveetha University, Chennai 600077, India; 4 Division for Globalization Initiative, Liaison Center for Innovative Dentistry Graduate School of Dentistry, Tohoku University, Sendai 980-8575, Japan; 5 Pediatric Dentistry, Dr. Sulaiman Al Habib Hospital, Ar Rayyan, Riyadh 14212, Saudi Arabia

**Keywords:** vestibular shield, orthodontics, oral habit, functional appliance

## Abstract

This systematic literature search was performed to determine the clinical effectiveness of vestibular shields (VSs) in children undergoing orthodontic treatment. A comprehensive electronic search was performed in May 2022 using three databases (Medline via PubMed, the Cochrane Central Register of Controlled Trials, and Ovid). The initial screening of articles was based on titles and abstracts. Studies meeting the inclusion criteria were retrieved for the final quality assessment and the methodological qualities were assessed according to the Newcastle-Ottawa Scale and Cochrane risk of bias. The initial search identified 262 publications, of which 15 studies were potentially eligible, with excellent intra-examiner reliability (K = 0.88). A total of five studies were selected for the final analysis, including one randomized controlled trial, three clinical trials, and one cohort study. VS may have potential impacts on orthodontic treatment, lips and dental arches, but further high-quality studies are warranted

## 1. Introduction

Vestibular shields (VSs) have been used in orthodontics over many decades now [1,2] and VS was first introduced to correct upper incisor protrusions. A VS is used to control interferences in dento-facial growth associated with abnormal lip and tongue function in the mixed dentition period [3]. It has been suggested from case reports that a VS can eliminate oral dysfunctions, change tooth position, and establish muscular balance [4,5]. The lip bumper (LB) effect on mandibular teeth has been reported by several authors in various parts of the world. The use of an LB results in a slight proclination of the incisors and a substantial widening of the canine and premolar region [6,7,8,9]. The effect of VSs on first molar distalization has been reported as negligible. Hence, it is understood that the arch length increases in the anterior region are only moderate [6,7,8,9]. An American study [6] used an LB and VS and found an additional distal movement and tipping of the first molar compared to an LB in the study population. These early positive findings with VSs motivated researchers to further research the VS and its effect on growing children. During early mixed dentition, most children have underdeveloped lips [10]. During this period, the growth of the lips is delayed compared to the growth of the face; eventually, they catch up to face growth late in the mixed dentition period. Lip exercises have been suggested during this period. In such children, various lip exercises have been reported to achieve good results [11,12,13,14]. On the other hand, the oral screen has also been reported as a suitable appliance for lip training exercises [15]. Prior studies provided specific data involving the effectiveness of VSs in orthodontic treatment, which indicated that vestibular screens could enhance the power of oro-facial muscles and change lip morphology and function in children [16,17,18]. In addition, VSs have also been used to deal with depraved oral habits such as mouth breathing, lower-lip sucking, and snoring [1,16,19,20,21,22]. Although the clinical benefits of VSs have been documented in various studies, there is no sufficient evidence in the published literature to support the use of VSs in patients with malocclusion. Therefore, the purpose of this scoping review was to find out if there was scientific evidence to support the clinical effectiveness of VSs in children undergoing orthodontic treatment.

## 2. Materials and Methods

### 2.1. Search Strategy

The national library of medicine (“Medline via PubMed), the “Cochrane central register of controlled trials,” and the Ovid^SP^ database were searched for relevant papers. All studies published up until May 2022 were included in the search. The reference lists in the selected studies were screened for additional papers. The details of the search terms are mentioned in PICO format (Table 1).

### 2.2. Eligibility Criteria

Studies were selected based on the following inclusion criteria:

1. Study design: clinical trials with controls and prospective and retrospective cohort studies of humans.

2. Participants: Children with good health in the dental transitional period.

3. Interventions: Treatment involving VSs alone or VSs in conjunction with the specialized training of lip muscles

4. Control: The use frequency and duration of VSs.

5. Outcome parameters: Changes in the lips, dental arch, incisors, and oral habits.

6. Only articles published in the English language were included.

### 2.3. Selection of Studies

Two independent reviewers screened the retrieved articles based on the inclusion criteria. The initial selection was based on titles and abstract reading. Those papers with titles and abstracts that suggested that they might be related to the objectives of the present review were planned for further full-text reading. The full texts of the final selected studies were used for data extraction and quality assessment, and disagreements between the two reviewers were resolved by group discussion.

### 2.4. Study Heterogeneity and Quality Assessment

**Figure 1:**
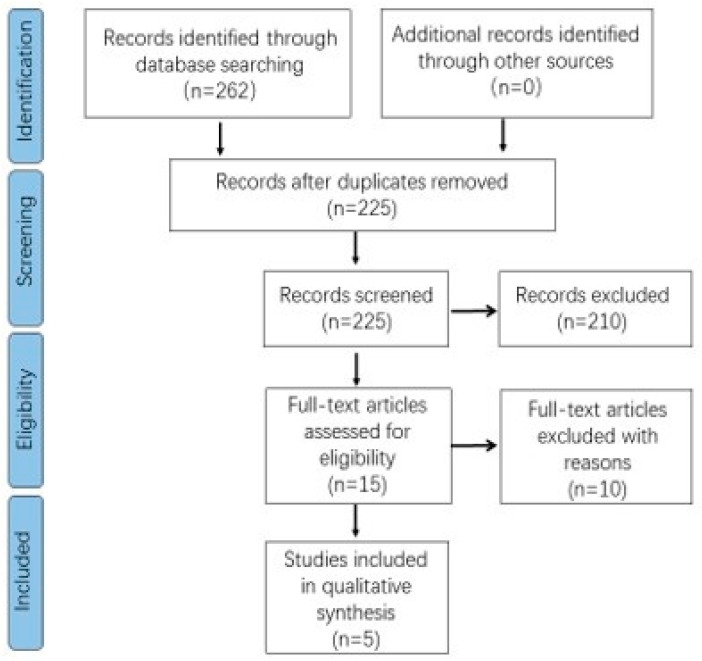
PRISMA flowchart showing the design of the search.

The study duration, participants’ demographic backgrounds including gender and age, interventions, outcome parameters, and conclusions were used to assess the heterogeneity of the selected studies. The search was limited to reviews published in the English language, and articles published up until May 2022 were included in the search. The present study protocol has been registered with the PROSPERO international prospective register of systematic reviews (PROSPERO: CRD42018108865) and was reported to adhere to the Preferred Reporting Items for Systematic Review and Meta-Analysis Protocols (PRISMA-P) statement [23]. The study review process was illustrated in Figure 1.

## 3. Results

### 3.1. Search and Selection Results

The search of the three databases retrieved 262 papers. A manual search of the reference lists in the selected studies did not retrieve any additional documents that met the criteria. Through the initial screening of the titles and abstracts, 210 articles were excluded from the analysis. A total of 15 papers were obtained and among them, 10 articles were ineligible for the final analysis. Finally, the selected studies were used for data extraction and quality analysis [3,18,19,24,25].

### 3.2. Study Characteristics

The review analyzed one randomized controlled trial [24], three clinical trials [3,18,24], and one retrospective cohort study [19]. The details of these five studies, such as the authors; year of publication; gender, number, and age of the subjects; treatment duration; and interventions, were summarized in Table 2. Two independent examiners were involved in the entire literature search, and kappa statistics were used for the intra-examiner reliability, which was excellent (K > 0.75).

### 3.3. Data Collection and Description

Based on the search, the total sample sizes of the five studies varied from 9 to 146, and the age range of the participants was between 6 and 15 years old. Only one study [19] did not mention the mean age of the patients. The gender of the participants was reported in all but one of the studies [19] without a gender ratio. The study periods ranged from 9 to 12 months among all the published studies on VSs in patients with malocclusion. Nevertheless, the duration of the study was not specified in an American study [19].

### 3.4. Treatment Outcomes

The treatment outcomes, including the outcomes for lip posture and dental arch (maxillary and mandibular) incisors, in the included studies are synthesized in Table 3.

#### 3.4.1. Lips

In all the included studies, lip changes due to VSs were reported, except for the study by Häsler and Ingervall [25]. Two studies [3,25] observed increased lip strength, and one study [8] stated that lip strength decreased after using VSs. Apart from lip strength, one study [25] reported increased lip height. Moreover, one of the studies [3] mentioned increased lip activity and another study [19] suggested that lip function was stimulated. Meanwhile, there was no significant difference in lip morphology and the use of VSs was evident.

#### 3.4.2. Dental Arch

The maxillary and mandibular dental arches were selected as general parameters to evaluate the effectiveness of VSs in all five studies [3,18,19,24,25] that mentioned the changes, and one study that did not provide specific data was excluded [15]. The width of the maxillary dental arch increased in three studies [18,24,25] and the length changes in the maxillary arch were varied. Among the rest, Häsler and Ingervall [25] noticed increasing lip length and a decrease in lip size was evident in Owman-Moll and Ingervall’s study [24]. In another study by Thüer and Ingervall [18], a decreased dental arch length that increased after treatment with a VS was reported. Changes in the mandibular dental arch were reported by only two studies [3,18] among the five available studies. One study [18] reported increased width but no changes in the length of the arches. Nonetheless, another study [3] found that only the length of the lower dental arch increased after using a VS.

#### 3.4.3. Incisors

Although all the studies mentioned considerable changes in the incisors, the outcomes differed significantly. In Owman-Moll and Ingervall’s study [25], the changes were evident in both the upper and lower incisors. The authors found that treatment using a VS brought ratiocination of the proclined maxillary incisors and proclination of the mandibular incisors. Thüer and Ingervall [18] did not observe a significant inclination of the maxillary incisors, whereas Häsler and Ingervall [25] documented a slight proclination of the incisors but the results were not statistically significant. Moreover, Tallgren et al. [3] also found slight retroclination in the maxillary incisors. Conversely, Toepfer et al. [19] observed that the incisor alignment worsened after using a VS in their study.

#### 3.4.4. Oral Habits

Only two studies [3,19] observed changes in oral habits with VSs in their study populations. Tallgren et al. [3] observed that the use of a VS had less of an influence on the habits of sucking and swallowing. However, Toepfer et al. [19] reported that a VS aided in the re-establishment of nasal breathing in habitual mouth-breathers. 

## 4. Discussion 

A vestibular shield is a functional appliance in orthodontic treatment [24,26,27] and has become a valuable method for dealing with many oral problems [28,29]. A total of five studies involving 225 participants were considered for the final analysis. Among these studies [3,18,19,24,25], lip changes, including lip height, lip strength, lip activity, and lip function, were mentioned as treatment outcomes. Owman-Moll and Ingervall [24] observed an increase in lip height and strength. Nevertheless, there was no conclusive evidence proving the influence on lip morphology in this study. The authors also concluded that the reason was the average growth of the participants, which means the significant difference could depend on the individuals. Two studies [3,19] also found changes in lip activity, whereas Thüer and Ingervall [18] observed initial increased activity of the lips and later, decreased activity. Thus, based on these studies, it was evident that using a VS may have a specific impact on the lip muscles.

Regarding maxillary/mandibular dental arches, all studies [3,18,19,24,25] observed changes in the width and length. Thüer and Ingervall [18] reported a relapse in the maxillary arch length, increasing the dental arch width. In addition, Häsler and Ingervall [25] concluded that using a VS could provide a more significant force. Although there were instabilities and limitations, some slight changes were noticed during the treatment duration in the dental arches using a VS. Five studies [3,18,19,24,25] reported changes in incisor position. Still, all the changes seemed very slight and the differences may be negligible. Only the American study [19] described the alignment of incisors worsening after treatment with a VS, which may have been due to the severity of malocclusion in the patients. The available data are insufficient to state the influence of VSs on oral habits. Nonetheless, two studies [3,19] focused on oral habits; however, the outcomes of these two studies differed. Toepfer et al. [19] observed the re-establishment of nasal breathing habits in children with mouth-breathing habits. Tallgren et al. [3] observed that the subjects had no significant changes in sucking and swallowing using the oral screen. An American study [19] opined that an oral screen is not a “universal appliance” but a beneficial device that can be used effectively in the mixed dentition period. Based on the included studies for the analysis, there is insufficient evidence that VSs can improve harmful oral habits.

The mixed conclusions reported from all five studies, including Owman-Moll and Ingervall’s analysis [24], reported that a VS had an additional effect compared with simple appliances, whereas Häsler and Ingervall [25] found that a VS was a greater force. Tallgren et al. [3] reported that a VS caused orofacial muscle hypotrophy. Toepfer et al. [19] opined that the oral screen was not a universal appliance but very useful. At the same time, Thüer and Ingervall [18] reported that the oral screen only had a limited duration of influence. The considerable heterogeneity in the outcomes may have resulted in a bias. The number of subjects in reported randomized controlled trials in the prospective and retrospective cohort studies was lower. The use of a VS during orthodontic treatment may change the width or length of the lips or the maxillary or mandibular dental arches. However, not all the reported studies provided specific data involving the deformity categories of the patients using VSs, which highlights the need for further studies. The impact of VSs on children with particular malocclusion also needs to be evaluated. The majority of the reports and studies focused on the effect of VSs on oral habits and lip forces; unfortunately, not many researchers studied the use and effect of VSs in growing children. There is still not enough information about VSs and their impact on lips, dental arches, and incisors. Large-scale multi-cantered clinical studies are essential to understanding VSs’ uses in orthodontic treatments. 

### Limitations

The review considered articles only in the English language, which is a potential limitation. The risk of bias in the selected studies may not be considered in a scoping review unlike a traditional systematic review, which is also a limitation. A scoping review may not provide a comprehensive evidence synthesis. However, a scoping review can provide basic information about the published studies. This is also considered a potential limitation.

## 5. Conclusions

Based on the quality analysis of the selected studies in the present review, the use of VSs during orthodontic treatment may positively impact the lips and dental arches of patients. Nevertheless, the specific effects of VSs are ambiguous, and further high-quality studies to evaluate the clinical effectiveness of VSs are recommended. 

## Figures and Tables

**Table 1:** MeSH terms and alternate terms were used in PICO format for the search.

Population	Intervention	Comparison	Outcome (s)
Children	Vestibular shield	-	Clinical effectiveness
Child	Orthodontic appliances	-	
Pediatric Paediatric Pedodontic Pedodontic Preschool Adolescent Teen Minor Young	Functional Herbst appliance Frankel Function regulator Oral screen	-	Mouth diseases Oral habits Orthodontic treatment Mouth breathing Snoring Lip muscles Tooth position Dental arch

**Table 2:** Characteristics of included studies in scoping review.

Authors	Year	Study Design	Duration	Participants		Age of Patients		Intervention
				Subjects	Gender	Mean	Range	
					M: F			
Toepfer et al [19]	1959	Retrospective observational study	-	146	-	-	7–15 Y	During the night
Owman-Moll and Ingervall [24]	1984	RCT	12 m	32	13:19	8.5 Y	6–13 Y	Oral screen during the night, lip-training exercise 10 min twice a day
Tallgren et al [3]	1998	CT	12 m	9	2:7	10.1 Y	7–12 Y	Oral shield during sleep
Thüer and Ingerval [18]	1990	CT (before and after)	9 m	16	10:6	9.25 Y	7–11 Y	Oral screen during the night, lip-training exercise 10 min during daytime
Häsler and Ingervall [25]	2001	CT (before and after)	12 m	22	7:15	10.5 Y	9–14 Y	Lip bumper day and night

RCT = Randomised control trial; m = months; M = male; F = female; Y = years.

**Table 3:** Summary of outcomes of included studies.

Studies	Outcomes					Conclusions
	Lip	Maxillary Dental Arch	Mandibular Dental Arch	Incisors	Oral Habits	
Owman-Moll and Ingervall [24]	Height and strength increased; morphology unchanged	Length decreased; width increased	No specific data	Proclined maxillary incisors retroclined; mandibular incisors proclined	No specific data	The oral screen has additional effects compared with a simple appliance
Häsler and Ingervall [25]	No specific data	Width increased; length increased	No specific data	Proclined slightly but not significant	No specific data	The vestibular shield has greater force
Tallgren et al. [3]	Activity increased	No specific data	Length increased	Maxillary incisors retracted slightly	No significant change in sucking and swallowing	Oral shield caused orofacial muscle decrease
Toepfer et al. [19]	Function promoted	No specific data	No specific data	Alignment worsened	Re-establishment of mouth breathing and sucking	The oral screen is not a universal appliance but is very useful
Thüer and Ingervall [18]	Strength increased but decreased later	Width increased; length decreased but increased later	Width increased; length unchanged	No significant inclination	No specific data	The oral screen has only a limited duration of influence

## Data Availability

Not applicable.
